# Overexpression of *OsNAR2.1* by *OsNAR2.1* promoter increases drought resistance by increasing the expression of *OsPLDα1* in rice

**DOI:** 10.1186/s12870-024-05012-9

**Published:** 2024-04-24

**Authors:** Yamei Wang, Hongyan Liu, Lu Bai, Ruifang Liu, Hongzhen Jiang, Jinfang Tan, Jingguang Chen

**Affiliations:** 1 School of Agriculture, Shenzhen Campus of Sun Yat-sen University, Shenzhen, Guangdong 518107 China; 2 School of Breeding and Multiplication (Sanya Institute of Breeding and Multiplication), Hainan University, Sanya, Hainan, 572025 China; 3 The High School Affiliated to Renmin, University of China, Shenzhen, Guangdong 518119 China

**Keywords:** Drought tolerance, Grain yield, *OsPLDα1*, *pOsNAR2.1:OsNAR2.1* expression, Rice, Water use efficiency

## Abstract

**Background:**

*pOsNAR2.1:OsNAR2.1* expression could significantly increase nitrogen uptake efficiency and grain yield of rice.

**Result:**

This study reported the effects of overexpression of *OsNAR2.1* by *OsNAR2.1* promoter on physiological and agronomic traits associated with drought tolerance. In comparison to the wild-type (WT), the *pOsNAR2.1:OsNAR2.1* transgenic lines exhibited a significant improvement in survival rate when subjected to drought stress and then irrigation. Under limited water supply conditions, compared with WT, the photosynthesis and water use efficiency (WUE) of transgenic lines were increased by 39.2% and 28.8%, respectively. Finally, the transgenic lines had 25.5% and 66.4% higher grain yield than the WT under full watering and limited water supply conditions, respectively. Compared with the WT, the agronomic nitrogen use efficiency (NUE) of transgenic lines increased by 25.5% and 66.4% under full watering and limited water supply conditions, and the N recovery efficiency of transgenic lines increased by 29.3% and 50.2%, respectively. The interaction between OsNAR2.1 protein and OsPLDα1 protein was verified by yeast hybrids. After drought treatment, PLDα activity on the plasma membrane of the transgenic line increased 85.0% compared with WT.

**Conclusion:**

These results indicated that *pOsNAR2.1:OsNAR2.1* expression could improve the drought resistance of rice by increasing nitrogen uptake and regulating the expression of *OsPLDα1*.

**Supplementary Information:**

The online version contains supplementary material available at 10.1186/s12870-024-05012-9.

## Background

Rice (*Oryza sativa* L.) is the main food for more than half of the world’s population. Drought stress is an important limited factor for plant growth, development and agricultural production [1, 2]. Drought stress can seriously impair the growth and development of many soil plants and is often responsible for large reduction in crop yields globally [3]. In addition, drought stress also can decrease the photosynthetic rate, restrict plant growth, and reduce crop yield [4]. Studies showed that drought stress could reduce seed setting rate and grain number per panicle, thereby causes rice yield reduction [5]. Therefore, improving the adaptability of plants to drought stress is of great significance for increasing agricultural productivity [6].

Nitrogen (N) is an essential nutrient for plants, which affects all the processes of rice from metabolism to growth and development [7]. Water and nitrogen nutrition are two coupled physiological processes that interact with each other. Nitrogen deficiency increased stomatal sensitivity to drought [7, 8]. Nitrogen participates in maintaining the physiological function of photosynthetic apparatus by increasing chlorophyll content and enhancing stomatal regulation [3]. Drought and nitrogen deficiency could significantly reduce net photosynthetic rate and Rubisco activity of plants, but drought is not able to affect Rubisco activity under sufficient nitrogen supply [8–10].

Phospholipase D (PLD) is a major lipid-degrading enzyme that hydrolyses phospholipids to produce phosphatidic acid (PA) and free polar head groups [11]. PLD not only affects the structure, function and stability of cell membranes by hydrolyzing phospholipids in cell membranes, but also plays vital role in cytoskeleton assembly, cell division, transmembrane transport, secretion and defensive response [12–17]. It was found that PLD could improve plant resistance to various stresses, including drought, salinity, freezing, and nutrient deficiency [18–21].

Nitrate assimilation related family (NAR2) is a small molecule protein, that are not known to possess any transport activity. Some highly affinity nitrate transporters belonging to the NRT2 family have been verified to be required to bind to NAR2 to perform their functions [7]. Quesada et al. [18]. first identified a small protein CrNar2 encoding about 200 amino acids, which has no known transporting activity but can replenish Chlamydomonas mutants lacking nitrate transporting function. The expression system of Xenopus oocyte confirmed that CrNar2 is a partner protein of its high affinity nitrate transporter [22]. The interaction between ATNAR2.1 and ATNRT2.1 in *Arabidopsis thaliana* was also demonstrated by oocyte expression and yeast split-ubiquitin systems [23]. Further studies showed that AtNAR2.1 participated in the control of AtNRT2.1 localization to the plasma membrane [24]. AtNAR2.1 and AtNRT2.1 polypeptide directly interacted on the plasma membrane to form an oligomer as a functional unit of high affinity nitrate influx in *Arabidopsis thaliana* roots [25]. In rice, OsNRT2.1, OsNRT2.2, and OsNRT2.3a also need to interact with OsNAR2.1 for nitrate uptake [26–28].

Yan et al. [27]. demonstrated that *OsNAR2.1* plays a key role in enabling the plant to cope with a variable nitrate supply. In addition, co-overexpression of OsNAR2.1 and OsNRT2.3a increased agronomic nitrogen use efficiency (NUE) in transgenic rice [28, 29]. Jiang et al., reported that the *OsAMT1.1* expression by nitrate-inducible promoter of *OsNAR2.1* increases NUE and rice yield [29]. In addition, *OsNAR2.1* interaction with *OsNIT1* and *OsNIT2* functions in root-growth responses to nitrate and ammonium [30, 31].

Our previous work extensively investigated the regulation of rice yield, drought tolerance, and NUE by the *OsNAR2.1*. It was found that the expression of *pOsNAR2.1:OsNAR2.1* could enhance rice field yield and NUE. Further analysis revealed that this enhancement primarily occurred through elevated expression of *OsNRT2.1*, thereby improv rice NUE [32]. Through the use of *pUbi: OsNAR2.1* and *OsNAR2.1* RNAi transgenic lines, we also discovered that *OsNAR2.1* is involved in the regulation of rice drought tolerance [32–34]. Overexpression of *OsNRT2.1* or *OsNRT2.3a* alone did not enhance rice drought tolerance, *OsNAR2.1* does not enhance rice nitrogen absorption or contribute to drought resistance by modulating the expression of *OsNRT2s*.

However, the specific mechanism by which OsNAR2.1 is involved in rice drought resistance remains unclear and requires further elucidation [30, 31]. Therefore, we have been investigating the regulatory mechanism of *OsNAR2.1* in rice drought tolerance. Several studies have previously indicated that *PLDα* could enhance plant resistance to various environmental stresses, including mechanical injury, cold injury, salt injury, and drought stress [21]. Thus, to further study the gene function of *OsNAR2.1*, we obtained some proteins that may interact with OsNAR2.1, including OsPLDα1 protein, by immunoprecipitation (Co-IP) and LC-MS/MS analysis. In this study, we confirmed that the interaction between OsNAR2.1 protein and OsPLDα1 protein, *pOsNAR2.1:OsNAR2.1* expression can improve the drought resistance in rice by increasing nitrogen uptake and regulating *OsPLDα1*.

## Results

### Drought stress sensitivity of *pOsNAR2.1:OsNAR2.1* transgenic lines at the seedling stage

**Fig. 1:**
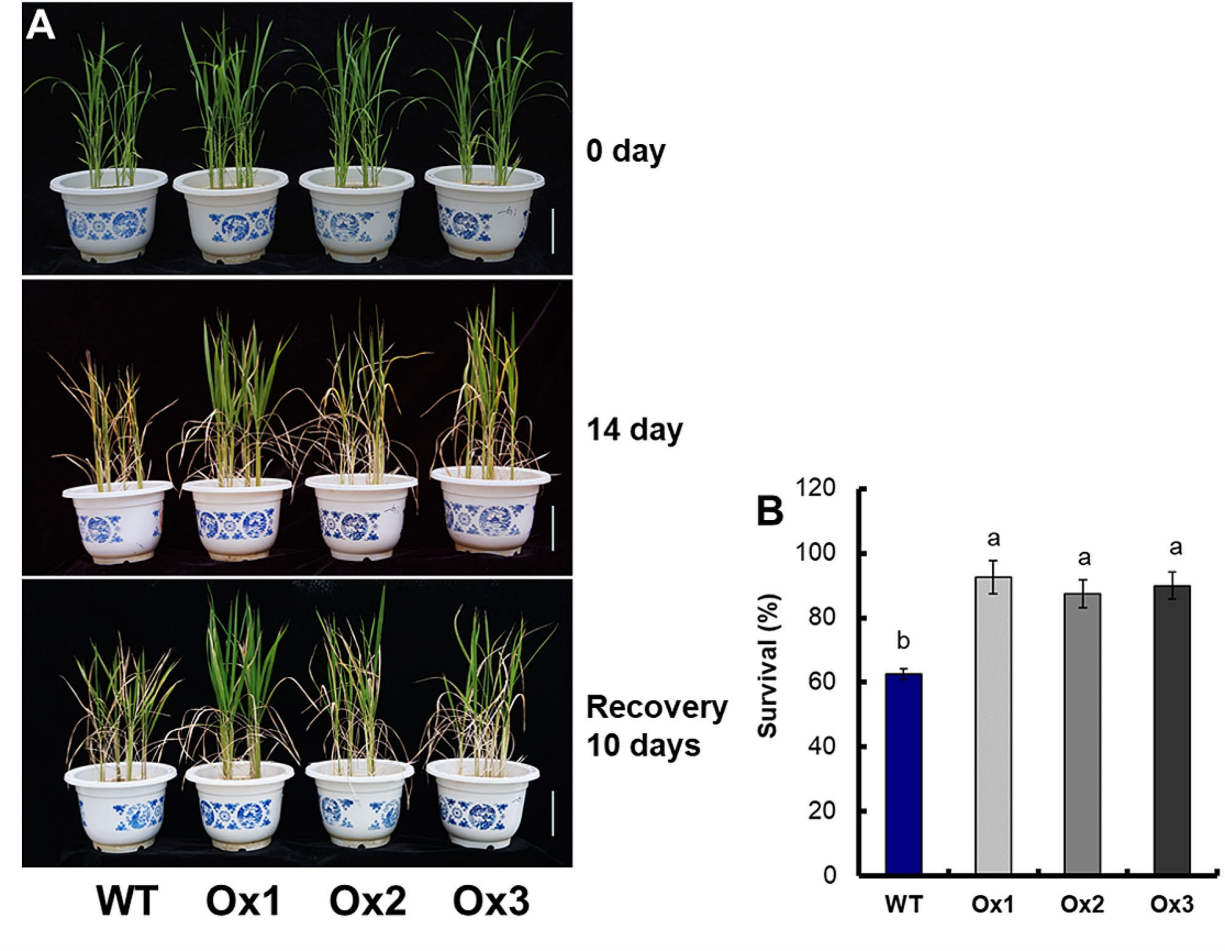
Drought stress sensitivity of *pOsNAR2.1:OsNAR2.1* transgenic lines at the seedling stage. (**A**) Phenotype of drought-stressed plants followed by recovery. The seedlings were grown for 21 days under well-watered conditions by maintaining 10 equal-sized seedlings per pot. Irrigation was withheld for 14 days, followed by rewatering for 10 days. Bar = 10 cm. (**B**) Seedling survival. After recovery, the number of seedlings with at least one fully expanded leaf was counted. Error bars: SE (*n* = 5). The different letters indicate a significant difference between the transgenic line and the WT (*P* < 0.05, one-way ANOVA, least significance difference model)

Under treatment of 15% PEG6000, the expression of *OsNAR2.1* in shoots and roots of WT was 3.5 times higher than those of the control (Fig. S1A). For transgenic lines, the expression of *OsNAR2.1* in drought was 4.8–5.1 times higher than those of the control (Fig. S1B). Irrigation was withheld for 14 days, followed by rewatering for 10 days (Fig. 1A), and the survival rate was measured. Approximately 62.6% of the WT recovered while 90.0% of the *pOsNAR2.1:OsNAR2.1* transgenic lines recovered (Fig. 1B).

### Physiological and biochemical under drought stress

**Fig. 2:**
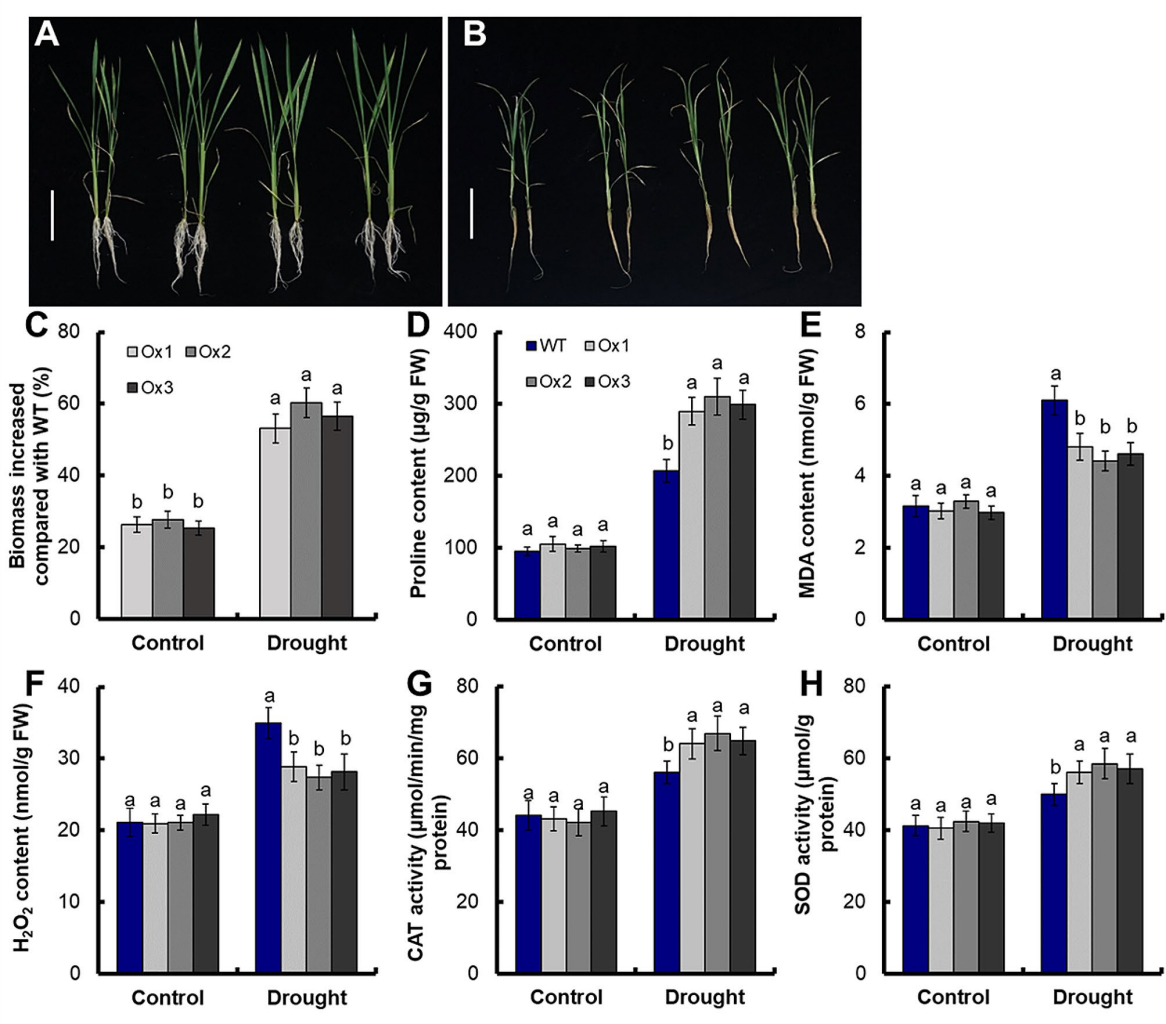
Physiological and biochemical changes at the seedling stage. Rice seedlings were grown with the normal IRRI solution for 2 weeks and then transferred to nutrient solution supplemented with 15% (w/v) PEG6000 for 2 weeks. Phenotype of transgenic lines grown in (**A**) control and (**B**) drought stress (15% PEG6000) conditions. Bar = 10 cm. (**C**) Increased biomass in transgenic plants relative to WT. (**D**) Proline content, (**E**) MDA content, (**F**) H_2_O_2_ content, (**G**) CAT activity and (**H**) SOD activity. Error bars: SE (*n* = 5). The different letters indicate a significant difference between the transgenic line and the WT (*P* < 0.05, one-way ANOVA, least significance difference model)

To further assess how *pOsNAR2.1:OsNAR2.1* expression affects rice growth in drought stress, the seedlings of transgenic lines were grown in normal IRRI solution for 2 weeks. Then they were transferred to nutrient solution without PEG6000 as a control (Fig. 2A) and nutrient solution supplemented with 15% (w/v) PEG6000 for 2 weeks (Fig. 2B). Under drought stress, wilting leaves of transgenic plants were less than WT (Fig. S2B), but there was no significant difference under the control conditions (Fig. S2A). Compared with control, the drought treatment led to suppression of root and shoot growth in all plants (Fig. S2D, S2E). Compared with WT, the biomass per plant of transgenic lines increased by 26.4% under control condition, and increased by 56.6% under drought stress (Fig. 2C, S2C). Additionally, significant differences were observed in the root/shoot ratio between WT and different transgenic plants in both control and treatment environments. Compared with WT, the root/shoot ratio per plant of transgenic lines increased by 7.4% under control condition, and increased by 22.9% under drought stress (Fig. 2C, S2F). There were no significant differences in proline content, Malondialdehyde (MDA) content, Hydrogen peroxide (H_2_O_2_) content, Catalase (CAT) activity and superoxide dismutase (SOD) activity between WT and transgenic lines under control conditions (Fig. 2D,2H). Compared with WT, the proline content of transgenic lines increased by 44.8% (Fig. 2D), the MDA content and H_2_O_2_ content of transgenic lines decreased by 24.3% and 19.5% (Fig. 2E F), the CAT activity and SOD activity of transgenic lines increased by 16.5% and 14.6% (Fig. 2G and H).

### Growth parameters under limited water supply conditions at anthesis stage

**Fig. 3:**
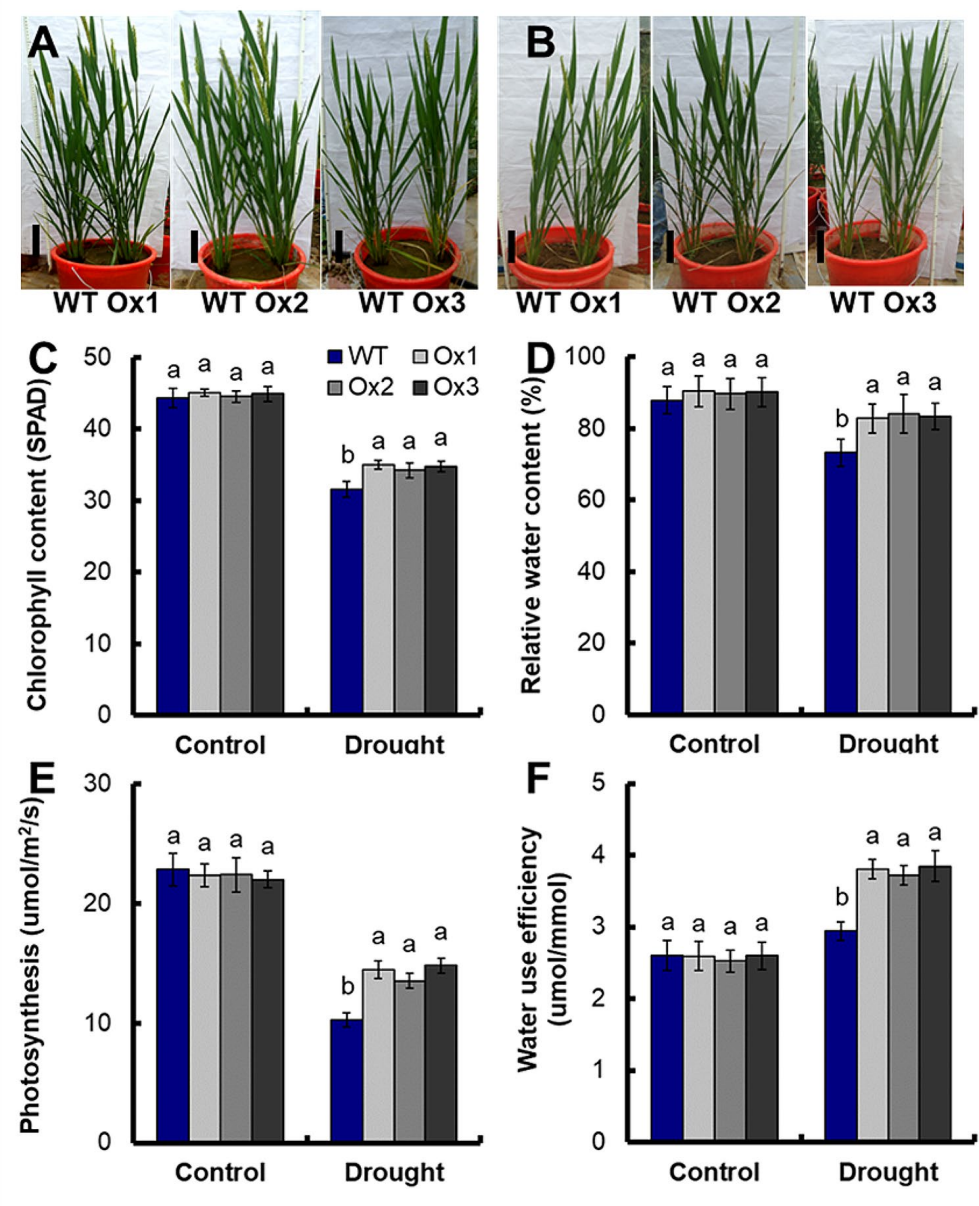
Growth parameters under limited water supply conditions. Phenotype of transgenic lines grown in (**A**) full watering (100% field capacity, Control) and (**B**) limited water supply conditions (60% field capacity, Drought) at anthesis stage. (**C**) Chlorophyll content, (**D**) relative water content, (**E**) photosynthesis rate and (**F**) water use efficiency were assayed. Error bars: SE (*n* = 5). The different letters indicate a significant difference between the transgenic line and the WT (*P* < 0.05, one-way ANOVA, least significance difference model)

Detailed analysis of drought response was conducted by limited water supply treatment, maintaining plants at 60% field capacity (Fig. 3B). There were no significant differences in chlorophyll content, relative water content, photosynthesis rate and water use efficiency (WUE) between WT and transgenic lines under full watering conditions at anthesis stage (Fig. 3C, D, E and F). Under limited water supply conditions, compared with WT, the chlorophyll content, relative water content, photosynthesis and WUE of transgenic lines increased by 9.7%, 13.9%, 39.2% and 28.8% (Fig. 3C, D, E and F). Chlorophyll content, relative water content and photosynthesis of WT under full watering conditions (Fig. 3A) was significantly higher than that under limited water supply conditions (Fig. 3B) at anthesis stage (Fig. 3C, D and E). However, the WUE of WT under full watering conditions was significantly lower than that under limited water supply conditions (Fig. 3F).

### Grain yield and NUE under limited water supply condition

**Fig. 4:**
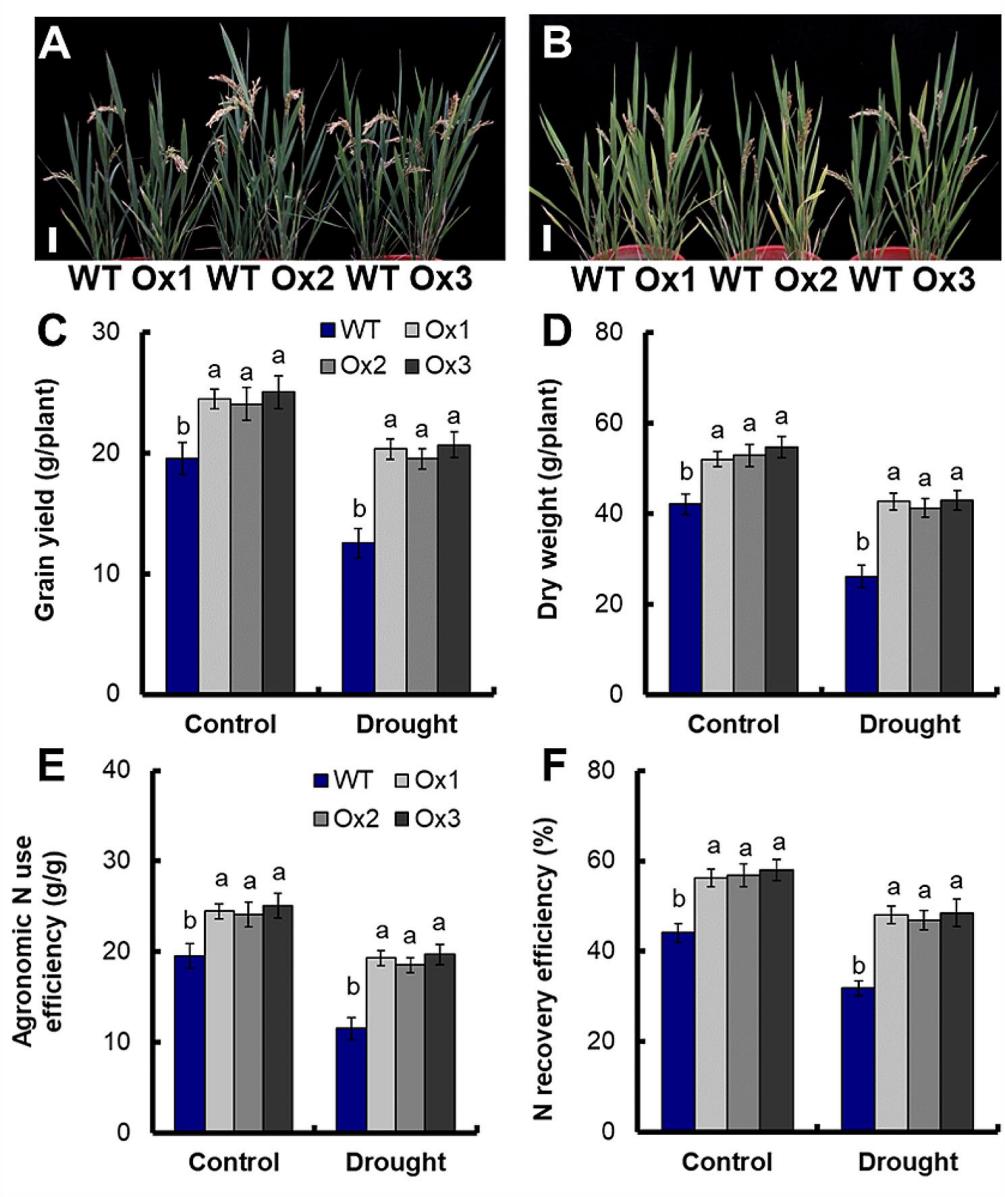
Grain yield and NUE under limited water supply conditions. Phenotype of transgenic lines grown in (**A**) full watering and (**B**) limited water supply conditions at maturity stage. (**C**) Grain yield, (**D**) dry weight, (**E**) agronomic N use efficiency and (**F**) N recovery efficiency were measured. Error bars: SE (*n* = 5). Bar = 10 cm. The different letters indicate a significant difference between the transgenic line and the WT (*P* < 0.05, one-way ANOVA, least significance difference model)

We analyzed the agronomic traits of WT and transgenic lines under full watering (Fig. 4A) and limited water supply conditions (Fig. 4B) at maturity stage. Compared with full watering conditions, seed setting rate, grain yield and total tiller number per plant of WT decreased by 37.2%, 41.1% and 16.7% under limited water supply conditions (Fig. S3D, 4C, S3A). Compared with WT, the total tiller number per plant of transgenic lines increased by 21.7% under full watering conditions and 19.7% under limited water supply conditions (Fig. S3A), grain weight of transgenic lines increased by 11.2% under full watering conditions and 41.3% under limited water supply conditions (Fig. S3B), grain number per panicle of transgenic lines increased by 13.5% under full watering conditions and 20.0% under limited water supply conditions (Fig. S3C), and seed setting rate of transgenic lines increased by 12.4% under full watering conditions and 31.4% under limited water supply conditions (Fig. S3D). Compared with WT, the grain yield of transgenic lines increased by 25.5% and 66.4% under full watering and limited water supply conditions, and the dry weight of transgenic lines increased by 26.6% and 61.7% respectively (Fig. 4C and D). In addition, compared with WT, the agronomic NUE of transgenic lines increased by 25.5% and 66.4% under full watering and limited water supply conditions, and the N recovery efficiency of transgenic lines increased by 29.3% and 50.2%, respectively (Fig. 4E and F).

### Protein and protein interaction between OsNAR2.1 and OsPLDα1

**Fig. 5:**
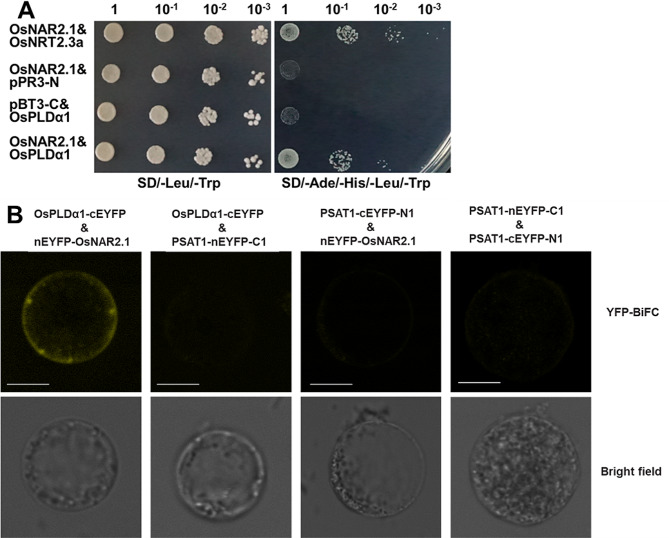
Protein and protein interaction between OsNAR2.1 and OsPLDα1. **A**: Testing the interaction of OsNAR2.1 and OsPLDα1 using the DUAL pairwise interaction kit with ADE2 and HIS3 as reporter genes. Yeast strain NMY51 carried each pair of bait and prey plasmid, co-expression of OsNAR2.1 and OsNRT2.3a as a positive gene control for protein interactions (Yan et al., 2011; Liu et al., 2014), pBT3-C and pPR3-N are the control vectors with no cloned cDNA. Cells grown on selective SD/-Ade/-His/-Leu/-Trp or control SD/-Leu/-Trp medium. **B**: Bimolecular fluorescent complementary assays to detect the interaction between OsPLDα1 and OsNAR2.1. The ORFs of OsPLDα1 and OsNAR2.1 were constructed on the PSAT1-cEYFP-N1 vector and PSAT1-nEYFP-C1 vector, respectively. Fluorescence could only be detected when OsPLDα1-cEYFP was combined with nEYFP-OsNAR2.1. Bars, 10 μm

OsNAR2.1 protein was fused to the C-terminal half of ubiquitin (Cub) and the artificial transcription factor LexA-VP16, and the OsPLDα1 proteins was fused to the mutated N-terminal half of ubiquitin (NubG). If the OsNAR2.1 and OsPLDα1 interaction resulted in reconstitution of the split ubiquitin (Nub and Cub), the reporter genes (HIS3 and ADE2) would allow the yeast to grow on selective medium (SD/-Ade/-His/-Leu/-Trp). Our data showed that positive control could grow normally on SD/-Ade/-His/-Leu/-Trp medium, indicating that OsNAR2.1 interacted with OsNRT2.3a, while negative control could not grow on SD/-Ade/-His/-Leu/-Trp medium, which was consistent with previous reports [27, 28]. The co-expression of OsNAR2.1 and OsPLDα1 could grow normally on SD/-Ade/-His/-Leu/-Trp medium (Fig. 5A), indicating that OsNAR2.1 and OsPLDα1 interacted.

Bimolecular fluorescent complementary assay (BiFC) was performed to clarify the interaction between OsNAR2.1 and OsPLDα1. In BiFC assays, the peptides nYFP and cYFP were combined to excite fluorescence only when nEYFP-OsNAR2.1 and OsPLDα1-cEYFP combined, otherwise there was no fluorescence excitation (Fig. 5B). These results indicated that an interaction existed between OsNAR2.1 and OsPLDα1.

### Drought resistance of *OsPLDα1* gene

To determine the effect of *OsPLDα1* on drought tolerance, *OsPLDα1* expressed in yeast strain CM52 was compared with the empty vector pYES2. The results showed that *OsPLDα1* could significantly rescue the growth of yeast strain CM52 when supplied with 20% PEG6000 or 2 M Sorbitol compared with *pYES2* (Fig. S4), suggesting that the expression of *OsPLDα1* could improve the drought tolerance of yeast.

### PLDα activity of WT and transgenic lines under drought stress conditions

**Fig. 6:**
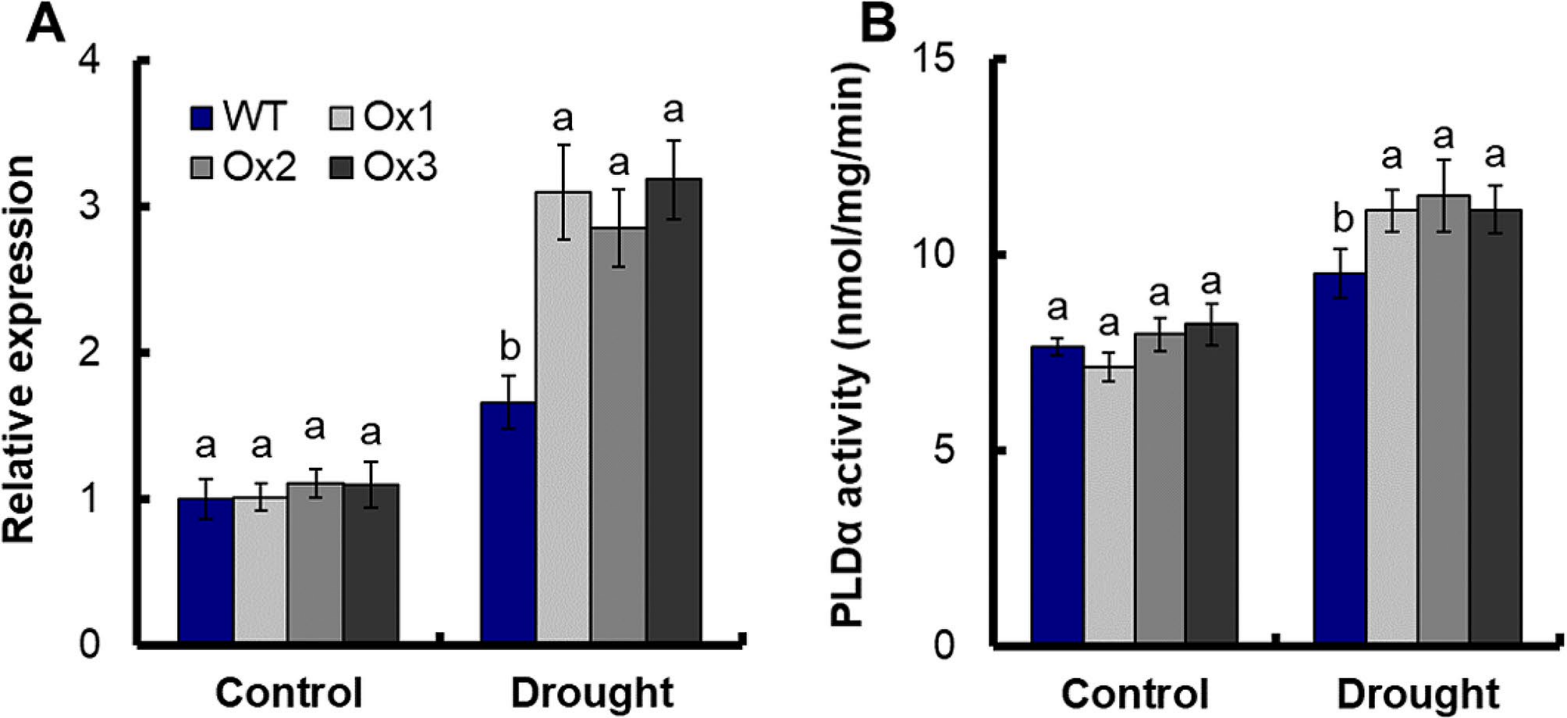
PLDα activity of WT and transgenic under drought stress conditions. Growth conditions and treatments were the same as described in Fig. 3. Samples from root of seedlings under control and drought stress (15% PEG6000) conditions. (**A**) Expression of *OsPLDα1* in the transgenic lines under drought stress conditions. Error bars: SE (*n* = 3). (B) PLDα activity of WT and transgenic under drought stress conditions. Error bars: SE (*n* = 5). The different letters indicate a significant difference between the transgenic line and the WT (*P* < 0.05, one-way ANOVA, least significance difference model)

**Fig. 7:**
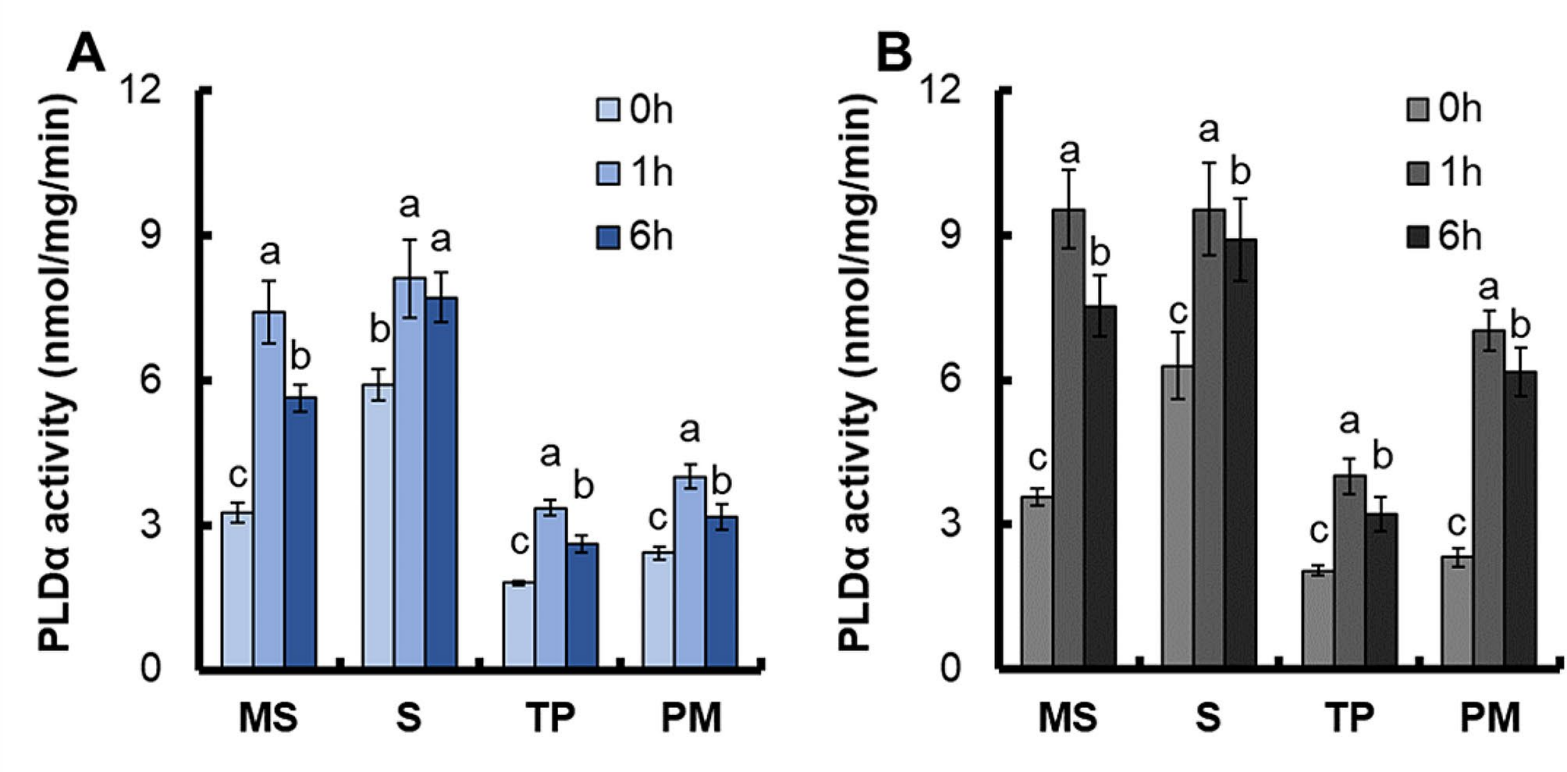
Changes of PLDα activities in the soluble and membrane fractions under drought stress conditions. Suspension Cells of (**A**) WT and (**B**) transgenic line Ox1. MS, membrane and soluble fractions, the protein from the supernatant after the centrifugation at 12 000 g; S, soluble fractions, from the supernatant after the centrifugation at 70 000 g; TP, toloplast vesicles; PM, plasma membrane vesicles. Error bars: SE (*n* = 5). The different letters indicate a significant difference between the transgenic line and the WT (*P* < 0.05, one-way ANOVA, least significance difference model)

We have analyzed the PLDα activity in roots of WT and transgenic lines. The expression of *OsPLDα1* in transgenic lines was not significantly different from that in WT under control conditions, but significantly increased under drought (Fig. 6A). Similarly, PLDα activity in transgenic lines was not significantly different from that in WT under control, but increased by about 18.5% under drought (Fig. 6B). After suspension cultured cells were treated with 15% PEG6000, the PLDα activities in soluble and membrane fractions were measured. After drought treatment, the PLDα activities of WT and transgenic line Ox1 increased in all fractions (Fig. 7A and B). Moreover, the increase of each fraction of PLDα activity in Ox1 was significantly higher than that of WT (Fig. 7A and B, S5). Compared with WT, the PLDα activity of Ox1 increased by 31.2% in membrane and soluble fractions (MS), 16.5% in soluble fractions (S), 21.5% in toloplast vesicles (TS) and 85.0% in plasma membrane vesicles (PM) after 1 and 6 h of drought treatment (Fig. S5).

## Discussion

Previous work extensively investigated the regulation of rice yield, drought tolerance, and NUE by the *OsNAR2.1* gene. It was found that the expression of *pOsNAR2.1:OsNAR2.1* could enhance rice field yields and NUE. Further analysis revealed that this enhancement primarily occurred through elevated expression of *OsNRT2.1*, thereby improving rice NUE (Chen et al., 2017). Through the use of *pUbi: OsNAR2.1* and *OsNAR2.1* RNAi transgenic lines, we also discovered that *OsNAR2.1* is involved in the regulation of rice drought tolerance. Overexpression of *OsNRT2.1* or *OsNRT2.3a* alone did not enhance rice drought tolerance, *OsNAR2.1* does not enhance rice nitrogen absorption or contribute to drought resistance by modulating the expression of *OsNRT2s*. However, the specific mechanism by which OsNAR2.1 is involved in rice drought resistance remains unclear and requires further elucidation (Chen et al., 2019). Therefore, we have been investigating the regulatory mechanism of *OsNAR2.1* in rice drought tolerance.

The experiment with drought treatment (withholding irrigation for 14 days) plus rewatering (10 days), showed the recovery rates of the *pOsNAR2.1:OsNAR2.1* transgenic lines (90%) were much higher than that of the WT (62.6%) (Fig. 1B). Compared with WT, the biomass of transgenic lines increased by 26.4% under control conditions, and increased by 56.6% under drought stress (Fig. 2C, S2C). Increasing the root/shoot ratio of plants can effectively enhance their adaptability to drought conditions. Under drought stress, transgenic plant lines exhibit significant higher root/shoot ratios compared to WT. Under drought stress treatment, compared with WT, the proline content, CAT activity and SOD activity of transgenic lines significantly increased (Fig. 2D, G and H) (*P* < 0.05), whereas the MDA content and H_2_O_2_ content of transgenic lines were significantly decreased (*P* < 0.05) (Fig. 2E and F). Proline is an important regulator of plant tolerance to various stresses such as drought and high salinity [35, 36]. CAT and SOD can decompose H_2_O_2_, the increase of CAT and SOD activity can reduce the concentration of H_2_O_2_ in cells, regulate reactive oxygen species (ROS) homeostasis, thereby improve plant resistance to stress [37, 38]. MDA content is an important index of cell damage in plant stress [37, 39, 40]. Under drought stress, the accumulation of H_2_O_2_ in rice increased, which can lead to cell damage and eventually result in cell death [39]. . These studies indicated that *pOsNAR2.1:OsNAR2.1* expression enhanced drought tolerance of transgenic rice seedlings.

Compared with WT, the the chlorophyll content, relative water content, photosynthesis and WUE of transgenic lines significantly increased under limited water supply(*P* < 0.05), (Fig. 3C, D, E and F), and there were no significant differences in chlorophyll content, relative water content, photosynthesis rate and WUE between WT and transgenic lines under full watering conditions (Fig. 3C, D, E and F). Stay-green can be an adaptation to drought, enabling the maintenance of carbon assimilation during grain filling. [41, 42], and as a consequence, improving the seed set and yield stability [43]. Chlorophyll, as a key pigment in photosynthesis, is able to absorb light energy and convert it into chemical energy, and it is essential for photosynthesis [44].Photosynthesis is a crucial source of biomass accumulation in all plants and is among the physiological processes most sensitive toabiotic stress [45]. The transgenic lines exhibited a 21.7% and 19.7% increase in total tiller number per plant (Fig. S3A), a 13.5% and 20.0% increase in grain number per panicle (Fig. S3C), a 12.4% and 31.4% increase in seed setting rate (Fig. S3D), and 25.5% and 66.4% increase in grain yield (Fig. 4C) under full watering and limited water supply, respectively. Drought significantly reduces the grain yield of rice [3]. Research has demonstrated that the main reason of rice yield reduction caused by drought stress is the decline in seed setting rate and grain number per panicle [5]. Drought stress seriously impacts the development of rice spikelet, leading to decreased seed setting rates and grain yield [46–48]. Drought stress also reduced the tiller number of rice [48]. Grain yield is the result of various interacting factors. The increase of photosynthesis, WUE, tiller number and seed setting rate ultimately resulted in the increase of grain yields of *pOsNAR2.1:OsNAR2.1* transgenic lines under limited water supply. These results indicated that *pOsNAR2.1:OsNAR2.1* expression could increase rice grain yield without causing adverse growth phenotypes under limited water supply conditions.Plant adaptation to drought involves efforts to absorb water from deeper soil layers and minimize water loss, and these responses involve a large number of genes and complex metabolic pathways [47, 49, 50]. N is a crucial nutrient for plants; influencing every aspect of rice from metabolism to growth and development of rice from metabolism to growth and development [51]. Nitrogen supply plays an important role in plant response to drought stress [1]. Under conditions of water stress, adjusting nitrogen supply can enhance crop adaptability to water stress by i optimizing water relationship [1, 10]. Ammonium nitrogen nutrition enhances the photosynthetic rate of rice under water stress during the early stage of development [3], and ammonium increases the tolerance of rice seedlings [7, 52–54]. Luo et al. [54]. reported overexpression of nitrate transporter gene *OsNRT2.1* increased yield and manganese accumulation in rice under alternating wet and dry condition [54]. Our previous results suggested that the influx rate of ^15^NH_4_^15^NO_3_ in the *pOsNAR2.1:OsNAR2.1* transgenic lines increased about 20% in 1.25 mM ^15^NH_4_^15^NO_3_^30^. Compared with WT, the agronomic NUE of transgenic lines increased by 25.5% and 66.4% under full watering and limited water supply conditions (Fig. 4E), and the N recovery efficiency of transgenic lines increased by 29.3% and 50.2% respectively (Fig. 4F). The improvement of nitrogen uptake by *pOsNAR2.1:OsNAR2.1* expression may be the reason for the significant improvement of drought tolerance.

The interaction between OsNAR2.1 protein and OsPLDα1 protein was verified by yeast hybrids (Fig. 5A) and BiFC (Fig. 5B). The expression of *OsPLDα1* and PLDα activity in *pOsNAR2.1:OsNAR2.1* transgenic lines was not significantly different from that in WT under control conditions, but increased under drought conditions (Fig. 6A and B). PLDα1-PA regulates abscisic acid (ABA) signaling, since PA binds ABI1, a negative regulator in ABA signaling, and inhibits the ABI1 repression [52], mediating ABA-promoted stomatal closure [54].

In *Arabidopsis thaliana*, the overexpression of PLDα1 resulted in heightened sensitivity to ABA, leading to enhanced stomatal closure, thereby reducing water loss and increasing drought tolerance [55–57]. Conversely,, Arabidopsis with a deficiency in AtPLDα1 exhibited increased water loss by transpiration [11]. Stomatal closure contributes to the increase in reactive oxygen species (ROS) [58], promotes osmotic regulation and helps plants adapt to drought conditions [59]. Overexpression of *OsPLDα1* increases drought tolerance of rice by maintaining the integrity of photosynthesizer, *OsPLDα1* gene overexpression resulted in a reduction in production loss under severe water deficit, and has no adverse effect on rice growth and development [60]. We also found that expression of *OsPLDα1* increased drought tolerance in yeast (Fig. S4). After drought treatment, the PLDα activity of WT and transgenic line Ox1 increased in all fractions (Fig. 7A and B). Moreover, the increase of each fraction of PLDα activity in Ox1 was significantly higher than that of WT (Fig. 7A and B, S5). In *Arabidopsis thaliana*, NAR2.1 controls NRT2.1 localization to the plasma membrane [24]. OsNAR2.1 may also be involved in controlling the localization of OsPLDα1 to the plasma membrane in rice. The PLDα activity of transgenic line Ox1 increased more on the plasma membrane, and increased 85.0% compared with WT (Fig. 7A and B,S1). Under stress conditions, the distribution of PLDα in subcellular cells can be changed [61]. After bacterial challenge in resistant interactions, OsPLDα was clustered preferentially in plasma membrane adjacent to bacterial cells [60]. PLDα on plasma membrane increased significantly when leaves of castor bean or Arabidopsis were wounded [56, 57]. After treatment of rice suspension cells with elicitors, the OsPLDα protein content in rice plasma membrane increased significantly [61]. As a result, the expression level of *OsPLD* was enhanced and the PLDα activity on plasma membrane was increased, which further increased the adaptability of plants to stress environment [61]. The expression of *pOsNAR2.1:OsNAR2.1* on *OsPLDα1* regulation may be part of the reasons for significantly improving drought resistance in rice. Rice PLD mediates the production of signaling molecules phosphatidic acid (Phosphatidic acid, PA) through hydrolyzing phospholipids, resulting in the reorganization of components of biochemical membranes. Studies in Arabidopsis and rice indicate that PLD is involved in lipid metabolism and signal transduction processes in plants under various stress conditions, with significant biological effects [62].

## Conclusions

In the present study, we confirmed that *pOsNAR2.1:OsNAR2.1* expression could improve the drought resistance of rice by increase the nitrogen uptake significantly and regulate the interaction protein OsPLDα1 of OsNAR2.1 in rice. After drought treatment, the PLDα activity of transgenic line increased more on the plasma membrane, and increased 85.0% compared with WT. In addition, *pOsNAR2.1:OsNAR2.1* expression significantly increased grain yield and NUE as compared to WT and led to minimal growth defects under drought stress. These results indicated that *pOsNAR2.1:OsNAR2.1* expression is a promising strategy to improve abiotic tolerance, especially to drought in rice.

## Materials and methods

### Plant materials and growth conditions

The generation and basic molecular properties of *pOsNAR2.1:OsNAR2.1* transgenic lines (Ox1, Ox2 and Ox3) were previously described in Chen et al. [29]. . To determine the seedling survival under drought stress, the seedlings were grown for 21 days under well-watered conditions by maintaining 10 equal-sized seedlings per pot. Irrigation was withheld for 14 days, followed by rewatering for 10 days.

For hydroponic drought stress experiments, rice seedlings were grown with the normal IRRI solution (1.25 mM NH_4_NO_3_, 0.3 mM KH_2_PO_4_, 0.35 mM K_2_SO_4_, 1 mM CaCl_2_·2H_2_O, 1 mM MgSO_4_·7H_2_O, 0.5 mM Na_2_SiO_3_, 20 µM NaFeEDTA, 20 µM H_3_BO_3_, 9 µM MnCl_2_·4H_2_O, 0.32 µM CuSO_4_·5H_2_O, 0.77 µM ZnSO_4_·7H_2_O and 0.39 µM Na_2_MoO_4_·2H_2_O, pH 5.5) for 2 weeks and then transferred to nutrient solution supplemented with 15% (w/v) PEG6000 for 2 weeks. The hydroponic experiments were carried out in a growth room with a 14 h light (30 °C) (8:00–22:00)/10 h dark (22 °C) (22:00–8:00) and 60% relative humidity. In all treatments, nutrient solutions were replaced every 2 days.

For limited water supply experiment, the rice plants, were cultivated in pots at the Experimental Station of the Nanjing Agricultural University, Nanjing, Jiangsu, China. Each pot contains 12 kg of soil with the following key chemical properties: organic matter, 12.11 g/kg; total N content, 0.88 g/kg; available P content, 94.54 mg/kg; exchangeable K, 178.50 mg/kg; and pH 6.52. Urea fertilization was applied at 3 stages with a total dosage of 2 g N/pot. Each genotype was set up with three replicates, with each replicate consisting of eight pots, randomly placed. Each pot contained one WT and one transgenic plant. Each pot had 1 WT and 1 transgenic seedling. All plants were normally irrigated within 4 weeks after transplanting. Then limited water supply (60% field capacity) and full watering (100% field capacity, control) were maintained throughout the experiment until completion of the life cycle.

### Gene expression analysis

Total RNA was extracted using TRIzol reagent (Vazyme Biotech Co, Ltd., People’s Republic of China). DNase I-treated total RNAs were subjected to reverse transcription (RT) with HiScript II Q Select RT SuperMix for qPCR (+ gDNA wiper) kit (Vazyme Biotech Co). Triplicate quantitative assays were performed using the 2×T5 Fast qPCR Mix (SYBRGreenI) kit (TsingKe Co, Ltd., People’s Republic of China). The primers for qRT-PCR were shown in Table S1.

### Proline content, MDA content, H_2_O_2_ content, CAT activity and SOD activity

The proline content was determined according to Kavi Kishor and Sreenivasulu [64]. MDA content was determined based on the method described by Shi et al. [65]. . CAT activity and SOD activity were measured as described by Ning et al. [66]. . H_2_O_2_ content was measured following the methods described by Mittler et al. [67]. .

### Chlorophyll content, relative water content, photosynthesis and WUE

The degree of relative chlorophyll content in the fully expanded last leaf was determined using an SPAD-502 Chlorophyll Meter (Minolta Co.). Relative water content was measured as described in the leaves for chlorophyll content measurements. Relative water content = (fresh weight - dry weight)/(turgid weight - dry weight) × 100% [38]. The same leaf was used to measure photosynthetic rate. Photosynthesis rate were measured in rice seedlings using a Li-COR6400 portable photosynthesis system equipped with a LED leaf cuvette (Li-COR, Lincoln, NE, United States). WUE was calculated using photosynthesis measurements and the transpiration rate. WUE = photosynthesis/transpiration rate [67].

### Total nitrogen content and NUE

Total N content was measured and NUE method for the calculation of the reference in Chen et al. [29]. . Agronomic NUE was calculated as grain yield / N supply; N recovery efficiency was calculated as total N accumulation at maturity / N supply.

### Protein-protein interaction assays

The interactions between OsNAR2.1 and OsPLDα1 proteins were tested using the DUAL membrane pairwise interaction kit (Dualsystems Biotech AG, Schlieren, Switzerland) [27, 28]. Full-length cDNAs of *NAR2.1* and *OsPLDα1* were cloned into pBT3-C (LEU2, KanR) and pPR3-N (TRP1, AmpR) expression vectors, respectively. The vectors were introduced into yeast strain NMY51 (MATa his3 trp1 leu2 ade2 LYS2::HIS3 ura3::lacZ ade2::ADE2 GAL4), respectively, according to the manufacturer’s protocol (Yeast Transformation Kit; Beijing Kulaibo Technology Co., Ltd), and transformants were selected on SD/-Leu/-Trp medium. Positive clones were cultured in SD/-Leu/-Trp liquid medium until the early logarithmic phase, concentrated and washed three times with sterile water. After sequential 10-fold dilutions, 8 µL of the cell suspension were spotted on SD/-Ade/-His/-Leu/-Trp medium, respectively. The plates were incubated at 30 °C for two days before the growth phenotypes were evaluated.

We visualized the interaction between OsPLDα1 and OsNAR2.1 using transient expression of split EYFP-labeled OsNAR2.1 and OsPLDα1 in rice blade protoplasts. We constructed C-terminal fusions of EYFP with OsNAR2.1 (nEYFP-OsNAR2.1) and N-terminal fusions of EYFP with OsPLD (OsPLDα1-cEYFP), and transformed protoplasts with both nEYFP-OsNAR2.1 and OsPLDα1-cEYFP. YFP was used as a reporter protein because color development occurred only when the two non-fluorescent fragments (nEYFP-OsNAR2.1 and OsPLDα1-cEYFP) interacted.

### Drought stress tolerance assays of *OsPLDα1* gene in yeast

The wild-type yeast strain CM52 was used as the host stain. Full-length cDNAs of *OsPLDα1* was cloned into *pYES2* expression vector [68]. Empty vector *pYES2* and *OsPLDα1-pYES2* vectors were introduced into CM52 cells, respectively, according to the manufacturer’s protocol (Yeast Transformation Kit; Beijing Kulaibo Technology Co., Ltd), and transformants were selected on Synthetic Dextrose medium without uracil (SD-Ura). Positive clones were cultured in SD-Ura liquid medium until the early logarithmic phase, concentrated and washed three times with sterile water. After sequential 10-fold dilutions, 8 µL of the cell suspension were spotted on SD-Ura medium without stress (Control), supplemented with 20% PEG6000 and supplemented with 2 M Sorbitol, respectively. The plates were incubated at 30 °C for three days before the growth phenotypes were evaluated.

### Membrane vesicle preparation and PLDα activity determination

Rice seeds were artificially shelled, sterilized with 75% ethanol and 5.5% NaClO, and rinsed repeatedly with sterile water. The treated seeds were seeded in induction medium (MS, 2.0 mg/L 2,4-D, 30.0 g/L sucrose, 8.0 g/L agar, pH = 5.8), and the callus was transferred to subculture medium seven days later. Callus with faster growth rate, no browning and better dispersion rate were selected and transferred to a 130 rpm shaking incubator for suspension growth, and 15% (w/v) PEG6000 was added to the medium for treatment (MS, 2.0 mg/L 2,4-D, 30.0 g/L sucrose, pH = 5.8).

Tonoplast vesicles and plasma membrane were isolated as described by Shen et al. [69]. . Briefly, the suspension cultured rice cells were homogenized in the buffer (250 mM mannitol, 30 mM HEPES-Tris, 0.5% brovine serum albumin, 3 mM ethylene glycol tetraacetic acid, 2 mM phenylmethanesulphonyl fluoride, 1 mM dithiothreitol, pH 7.0), and the homogenate was centrifuged for 10 min at 12,000 g and the supernatant was centrifuged for 30 min at 70,000 g. The sediment was re-suspended in 1 mL suspension buffer (250 mM mannitol, 2.5 mM Hepes‐Tris, 0.5 mM dithiothreitol, pH = 6.8). The fractions were layered on top of the suspension medium containing sucrose at 10, 25, 32, and 40% (w/w). After centrifugation at 100 000 g for 2 h, the 10/25% and 32/40% interfaces were collected and used as tonoplast and plasma membrane vesicles, respectively.

Phospholipase Dα activity was determined by Phospholipase D Activity Detection Kit (Shanghai Suoqiao Biotechnology Co, Ltd., People’s Republic of China).

### Statistical analysis

Data were analyzed by ANOVA using the SPSS 10 program (SPSS Inc., Chicago, IL, United States). The different letters indicate a significant difference between the transgenic line and the WT (*P* < 0.05, one-way ANOVA, least significance difference model).

### Electronic supplementary material

Below is the link to the electronic supplementary material.

Supplementary Material 1

Supplementary Material 2

Supplementary Material 3

Supplementary Material 4

Supplementary Material 5

Supplementary Material 6

## Data Availability

The phenotypic data of the current study is available in the Additional file 1: Table S1. Any other datasets used and/or analyzed are available upon request.

## Abbreviations

ABA: Abscisic acid
CAT: Catalase
Co-IP: Co-Immunoprecipitation
Cub: C-terminal half of ubiquitin
H_2_O_2_: Hydrogen peroxide
MDA: Malondialdehyde
MS: Membrane and soluble fractions
NAR2: Nitrate assimilation related family
ROS: Reactive oxygen species
SOD: Superoxide dismutase
