# Supplementary material for: Overexpression of OsNAR2.1 by OsNAR2.1 promoter increases drought resistance by increasing the expression of OsPLDα1 in rice
Source: BMC Plant Biol. 2024 Apr 24;24:321. doi: 10.1186/s12870-024-05012-9 (PMC11040742; doi:10.1186/s12870-024-05012-9)
Supplement: Supplementary file 1 — Supplementary Material 1 [file 12870_2024_5012_MOESM1_ESM.docx]

**Additional file 1: Table S1** Primers used for qRT-PCR

| Gene name | Forward primer (5'to3') | Reverse primer (5'to3') |
| --- | --- | --- |
| *OsActin* | GGAACTGGTATGGTCAAGGC | AGTCTCATGGATAACCGCAG |
| *OsNAR2.1* | GTCGTCGAGAAGCGCAAGA | GTCCACTGAAGCTGCGAACTT |
| *OsPLDα1* | TTGGTGGCCTTGATCTCTGT | TCATGCCATGGCTCTCTAGG |
