# Supplementary material for: Overexpression of OsNAR2.1 by OsNAR2.1 promoter increases drought resistance by increasing the expression of OsPLDα1 in rice
Source: BMC Plant Biol. 2024 Apr 24;24:321. doi: 10.1186/s12870-024-05012-9 (PMC11040742; doi:10.1186/s12870-024-05012-9)
Supplement: Supplementary file 2 — Supplementary Material 2 [file 12870_2024_5012_MOESM2_ESM.docx]

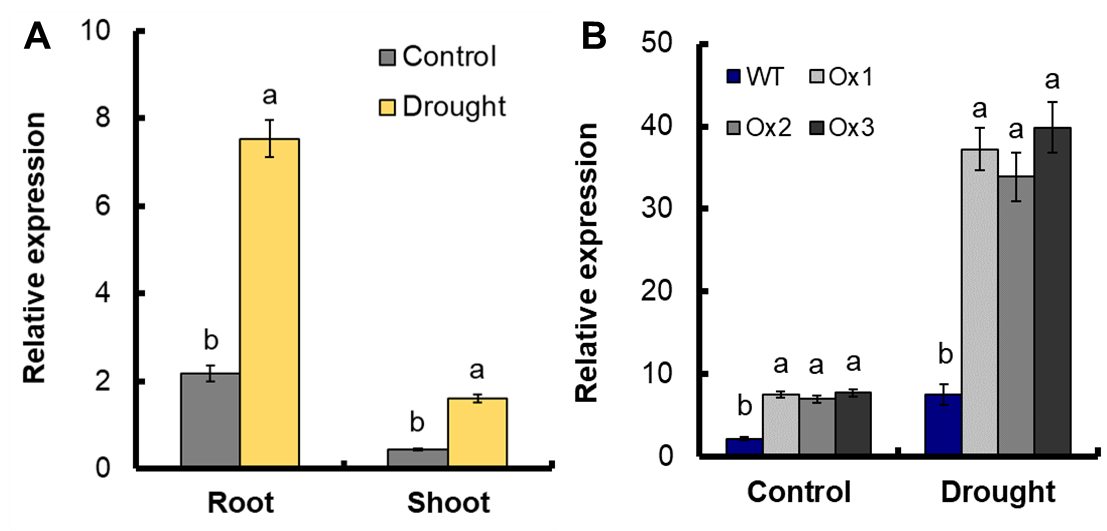


**Additional file 2: Figure S1** Expression pattern analysis of *OsNAR2.1* under drought stress treatment by real-time RT-PCR. Rice seedlings were supplied with normal IRRI solution (Control) or normal nutrient solution containing 15% (w/v) PEG6000 (Drought) for three days. RNA was extracted from roots and shoots of rice cv. *Wuyunjing7*. Error bars: SE (n = 3). The different letters indicate a significant difference between the control and drought (*P* < 0.05, one-way ANOVA, least significance difference model).
