# Supplementary material for: Overexpression of OsNAR2.1 by OsNAR2.1 promoter increases drought resistance by increasing the expression of OsPLDα1 in rice
Source: BMC Plant Biol. 2024 Apr 24;24:321. doi: 10.1186/s12870-024-05012-9 (PMC11040742; doi:10.1186/s12870-024-05012-9)
Supplement: Supplementary file 3 — Supplementary Material 3 [file 12870_2024_5012_MOESM3_ESM.docx]

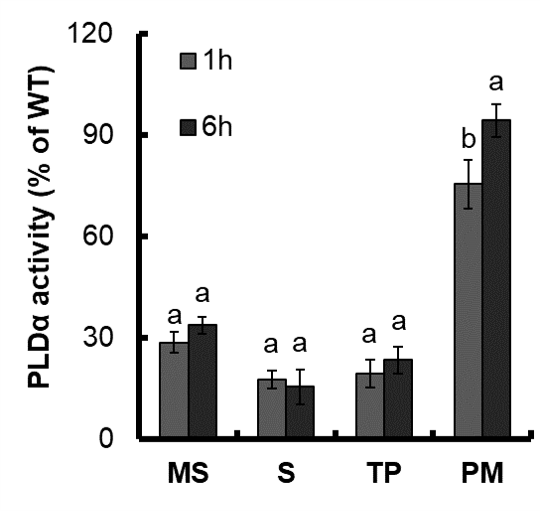


**Additional file 6: Figure S5** PLDα activities in the soluble and membrane fractions of transgenic lines was changed compared with WT under drought stress conditions. MS, membrane and soluble fractions, the protein from the supernatant after the centrifugation at 12 000 g; S, soluble fractions, from the supernatant after the centrifugation at 70 000 g; TP, toloplast vesicles; PM, plasma membrane vesicles. Error bars: SE (n = 5). The different letters indicate a significant difference between the transgenic line and the WT (*P* < 0.05, one-way ANOVA, least significance difference model).
