# Supplementary material for: Overexpression of OsNAR2.1 by OsNAR2.1 promoter increases drought resistance by increasing the expression of OsPLDα1 in rice
Source: BMC Plant Biol. 2024 Apr 24;24:321. doi: 10.1186/s12870-024-05012-9 (PMC11040742; doi:10.1186/s12870-024-05012-9)
Supplement: Supplementary file 4 — Supplementary Material 4 [file 12870_2024_5012_MOESM4_ESM.docx]

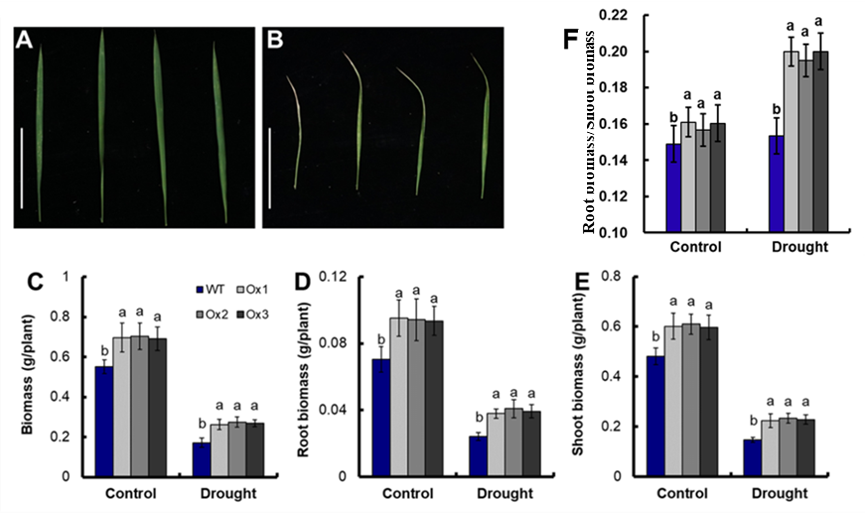


**Additional file 3: Figure S2** The biomass under drought stress conditions. Growth conditions and treatments were the same as described in Fig. 2. Photographs of leaves of seedlings under (A) control and (B) drought stress (15% PEG6000) conditions. Bar = 10 cm. (C) Total, (D) root, (E) shoot biomass (dry weight) and (F) root/shoot ratio of plants grown in control and drought stress conditions. Error bars: SE (n = 5). The different letters indicate a significant difference between the transgenic line and the WT (P < 0.05, one-way ANOVA, least significance difference model).
