# Supplementary material for: Overexpression of OsNAR2.1 by OsNAR2.1 promoter increases drought resistance by increasing the expression of OsPLDα1 in rice
Source: BMC Plant Biol. 2024 Apr 24;24:321. doi: 10.1186/s12870-024-05012-9 (PMC11040742; doi:10.1186/s12870-024-05012-9)
Supplement: Supplementary file 5 — Supplementary Material 5 [file 12870_2024_5012_MOESM5_ESM.docx]

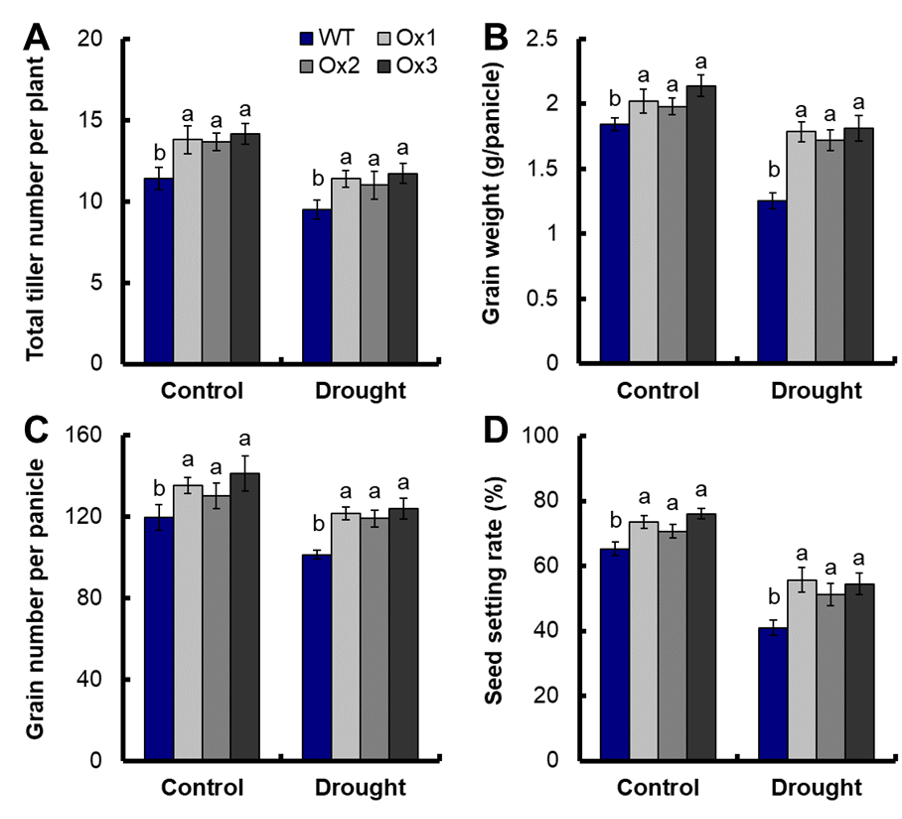


**Additional file 4: Figure S3** Agronomic traits under limited water supply conditions. Phenotype of transgenic lines grown in full watering (Control) and limited water supply conditions (Drought) at maturity stage. (A) Total tiller number per plant, (B) grain weight, (C) grain number per panicle and (D) seed setting rate were measured. Error bars: SE (n = 5). The different letters indicate a significant difference between the transgenic line and the WT (*P* < 0.05, one-way ANOVA, least significance difference model).
