# Supplementary material for: Overexpression of OsNAR2.1 by OsNAR2.1 promoter increases drought resistance by increasing the expression of OsPLDα1 in rice
Source: BMC Plant Biol. 2024 Apr 24;24:321. doi: 10.1186/s12870-024-05012-9 (PMC11040742; doi:10.1186/s12870-024-05012-9)
Supplement: Supplementary file 6 — Supplementary Material 6 [file 12870_2024_5012_MOESM6_ESM.docx]

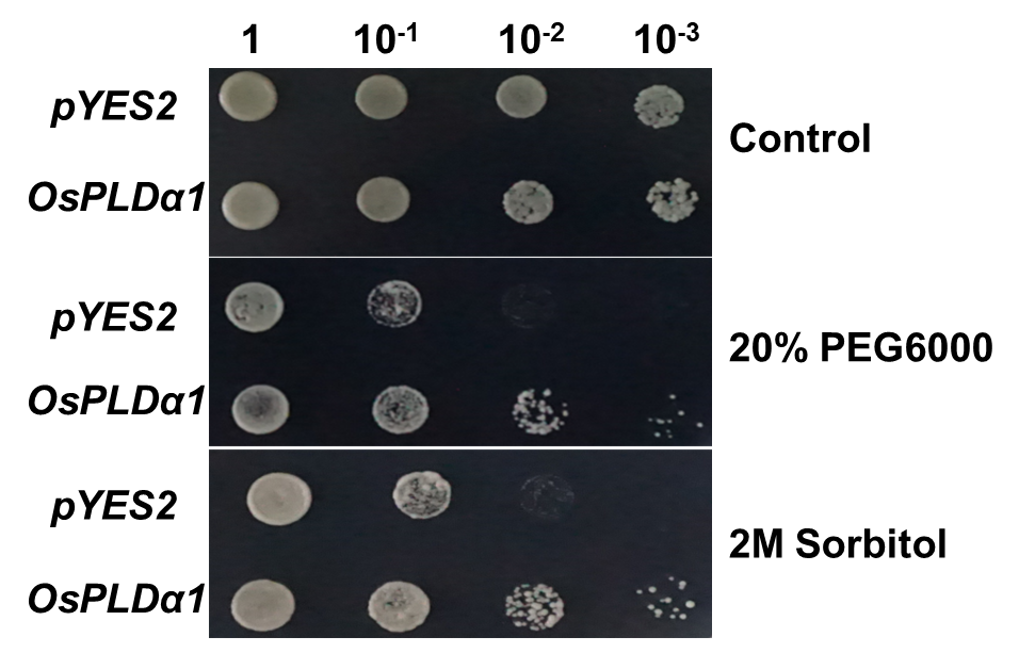


**Additional file 5: Figure S4** Effect of *OsPLDα1* in yeast against drought stress. *OsPLDα1* expressed in yeast strain CM52 was compared with the empty vector *pYES2*. Yeast cell density was adjusted to OD600 at 1.0. Serial dilutions of 1:10 transformed yeast cells were growing on solid SD-Ura medium without stress (Control), supplemented with 20% PEG6000 or 2 M Sorbitol at 30°C for three days.
